# The optimal stent‐strategy in paediatric pyeloplasty: a systematic review and network meta‐analysis

**DOI:** 10.1111/bju.70423

**Published:** 2026-08-16

**Authors:** Ophelia Aubert, Samira M'Tiri, Andreas Kühnapfel, David Petroff, Caelán Max Haney‐Aubert, Maria Moormann, Lily Skarba, Pia Erdoesi, Raimund Stein, Michael Boettcher, Martin Lacher

**Affiliations:** ^1^ Department of Pediatric Surgery University of Heidelberg, University Hospital Mannheim Mannheim Germany; ^2^ Department of Urology University of Heidelberg, University Hospital Mannheim Mannheim Germany; ^3^ Center for Pediatric, Adolescent and Reconstructive Urology University Medical Center Mannheim Heidelberg University Mannheim Germany; ^4^ Clinical Trial Centre Leipzig Leipzig University Leipzig Germany; ^5^ Department of Pediatric Surgery University Hospital Leipzig Leipzig Germany

**Keywords:** paediatric pyeloplasty, pelvi‐ureteric junction obstruction (PUJO), hydronephrosis, postoperative complications, urine leakage

## Abstract

**Objectives:**

To compare internal JJ stenting, external transanastomotic stenting and stentless repair after Anderson–Hynes pyeloplasty in children.

**Patients and Methods:**

A systematic review and network meta‐analysis of children with pelvi‐ureteric junction (PUJ) obstruction undergoing Anderson‐Hynes pyeloplasty. The MEDLINE, EMBASE, Cochrane Library and Web of Science databases were searched from inception to February 2025. Randomised and non‐randomised comparative studies were included. Network meta‐analysis compared JJ stenting, external stenting and no stenting. Risk of bias and certainty of evidence were assessed.

**Results:**

A total of 26 studies including 3298 children were analysed. Overall complications did not differ significantly between strategies and operative success was high. External stenting was associated with lower odds of major complications compared with no stenting (odds ratio [OR] 0.56, 95% confidence interval [CI] 0.33–0.93). The JJ and external stenting were associated with lower odds of major urine leakage or urinoma compared with stentless repair (OR 0.20, 95% CI 0.07–0.60; and OR 0.20, 95% CI 0.07–0.61, respectively). Minor complications were similar between strategies, as were stent‐related complications between JJ and external stenting. JJ stenting was associated with higher odds of urinary tract infection or pyelonephritis compared with no stenting, but with shorter hospital stay compared with external stenting. Certainty of evidence was low or very low for most outcomes.

**Conclusion:**

Paediatric pyeloplasty achieves high success rates regardless of drainage strategy. Stented approaches may reduce major leakage‐related complications compared with stentless repair, while JJ stents may increase infectious complications. Drainage strategy should remain individualised.

**Abbreviations**
GRADE: Grading of Recommendations, Assessment, Development and Evaluation
MD: mean difference
OR: odds ratio
PRISMA: Preferred Reporting Items for Systematic Reviews and Meta‐analyses
PUJO: PUJ obstruction
RCT: randomised controlled trial
RoB‐2: Cochrane Risk of Bias 2
ROBINS‐I: Risk Of Bias In Non‐randomised Studies of Interventions

## Introduction

Pelvi‐ureteric junction obstruction (PUJO) is one of the most common congenital anomalies of the urinary tract, with an incidence of approximately one in 1000–1500 live births and represents the most frequent cause of neonatal hydronephrosis [1, 2]. If untreated, PUJO may result in progressive hydronephrosis and deterioration of renal function [3]. Surgical correction is indicated in symptomatic patients, in children with impaired differential renal function at presentation, or when serial assessment demonstrates progressive hydronephrosis or deterioration of renal function. The Anderson–Hynes dismembered pyeloplasty remains the ‘gold standard’, with success rates of ~95% [4].

Over the past decades, management has shifted from open surgery toward laparoscopic and robot‐assisted techniques, which demonstrate comparable success rates while offering potential advantages such as reduced postoperative pain and improved cosmetic outcomes [5, 6]. Despite these advances, the optimal postoperative drainage strategy remains debated.

Currently, three drainage strategies are employed: internal JJ stenting, external transanastomotic stenting and stentless repair. Stenting aims to ensure drainage and protect the anastomosis, but each approach has trade‐offs regarding complication rate, postoperative care and patient and caregiver burden [7, 8, 9, 10, 11]. External stents may be associated with dislodgement, blockage or catheter‐related infection, whereas JJ stents require a second anaesthetic procedure for removal and may increase the risk of UTI or VUR [4, 12]. Stentless repair avoids stent‐related morbidity but raises concerns regarding urine leakage and urinoma formation [13, 14].

The most recent network meta‐analysis by Liu et al. [15] in 2019 included 15 comparative studies, of which four were randomised controlled trials (RCTs). It found no significant differences in operative success or overall complication rates among drainage strategies. However, the analysis was limited by a small cumulative sample size, limited randomised evidence, lack of separation between major and minor complications, and absence of structured certainty‐of‐evidence assessment.

Persistent uncertainty on the optimal drainage strategy and recently published data warrant an updated synthesis. In the present study, we conducted an updated network meta‐analysis comparing JJ stenting, external stenting and stentless pyeloplasty in paediatric patients. The primary outcome was overall complication rate, with secondary analyses of major and minor complications and specific postoperative events. By incorporating recent evidence within a structured network meta‐analytic framework, this study aimed to provide a comprehensive evaluation of postoperative drainage strategies in paediatric pyeloplasty.

## Patients and Methods

This systematic review and meta‐analysis was conducted according to a pre‐specified protocol registered in the International Prospective Register of Systematic Reviews (PROSPERO; CRD42025635956) and reported in accordance with the Preferred Reporting Items for Systematic Reviews and Meta‐analyses (PRISMA) guidelines (Appendix S7) [16].

### Search Strategy

A systematic literature search was conducted in MEDLINE (via PubMed), EMBASE, Cochrane Library and Web of Science from database inception until February 2025. The search strategy combined subject headings and free‐text terms related to the paediatric population, stent strategies and surgical treatment of PUJO. Search terms included variations of ‘child’, ‘pediatric’, and ‘infant’, as well as terms such as ‘stent’, ‘double J’, ‘DJ’ and ‘JJ’. Procedure‐related terms included ‘pyeloplasty’, ‘ureteropelvic junction obstruction’, ‘UPJO’ and ‘ureteral obstruction’. Database‐specific adaptations of the search syntax were applied as appropriate (Appendix S1).

Reference lists of relevant reviews and eligible studies were screened manually to identify additional publications. Clinical trial registries were reviewed to identify ongoing or unpublished studies and study authors were contacted when necessary. The search strategy was developed in collaboration with an experienced medical librarian.

All records were imported into EndNote for duplicate removal and management. Two reviewers independently screened titles and abstracts, followed by full‐text assessment according to pre‐defined eligibility criteria. Study selection was documented using a PRISMA flow diagram (Fig. 1).

**Fig. 1:**
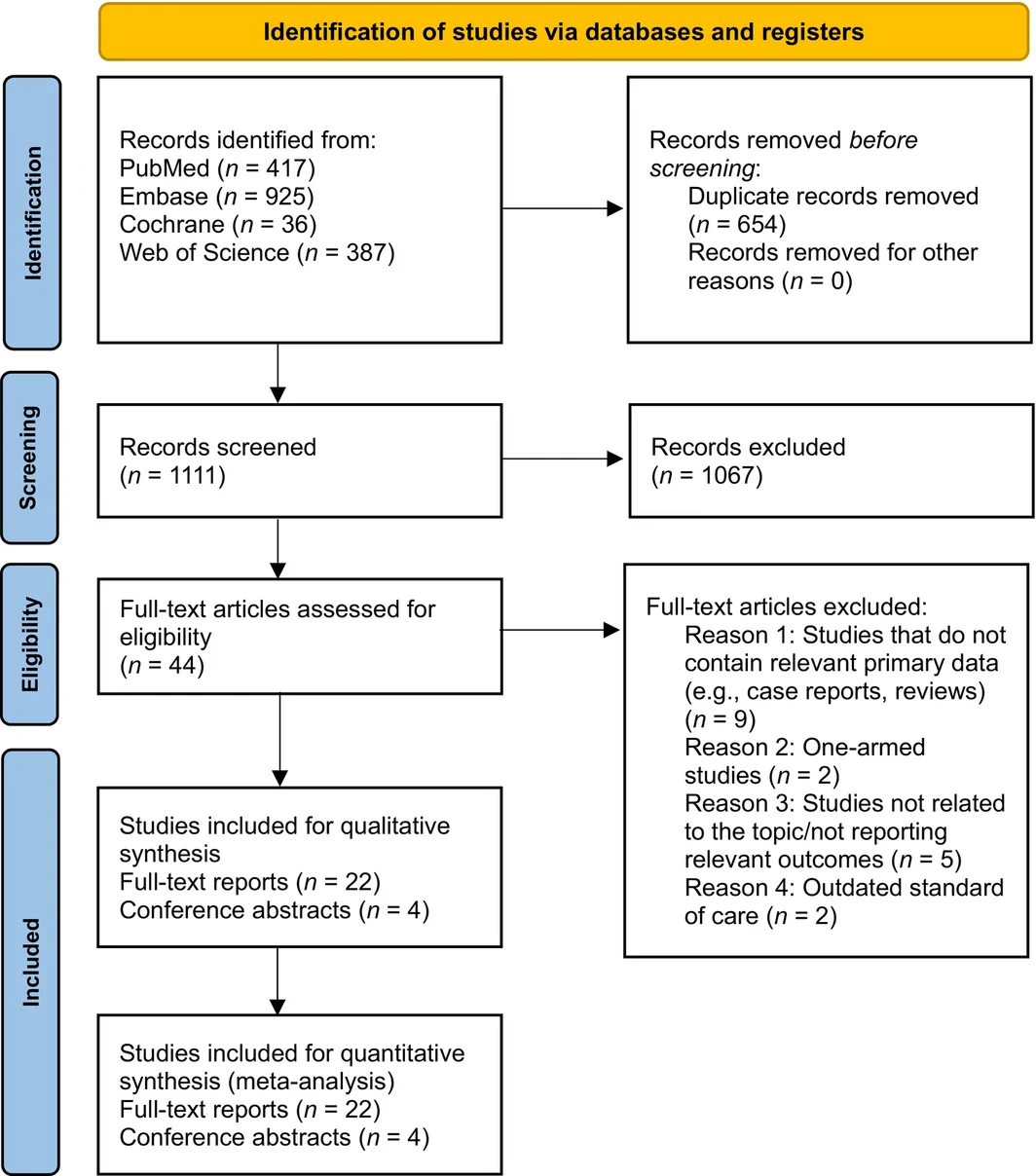
The PRISMA flowchart of the study selection process for the network meta‐analysis.

### Selection Criteria

Eligibility criteria were defined according to the PICO framework.

- Population (P): paediatric patients (aged <18 years) with PUJO undergoing Anderson–Hynes dismembered pyeloplasty
- Intervention (I): Anderson–Hynes pyeloplasty with internal JJ stent placement or external transanastomotic stent placement
- Comparator (C): Anderson–Hynes pyeloplasty without stent placement
- Outcomes (O): The primary outcome was the overall number of complications, as reported by the authors or calculated as the sum of all specific postoperative complications. Secondary outcomes included major and minor complications (according to the Clavien–Dindo classification) [17], operative success, defined as unobstructed urine outflow from the renal pelvis to the ureter or resolved/improved hydronephrosis on ultrasound or as defined by study, renal function, length of hospital stay (days) and operative time (min). Specific complications including urine leakage or urinoma stratified as major or minor complications, UTI, pyelonephritis, stent‐related complications, renal function, re‐stenosis and redo‐pyeloplasty were examined. Stent‐related complications included stent re‐insertion, stent migration or expulsion, inability of stent placement and stent blockage

Studies were eligible if they were RCTs, prospective non‐randomised studies, or retrospective comparative studies including at least 10 paediatric patients and reporting at least one pre‐defined outcome of interest. Eligible studies had to compare JJ stent placement, external stent placement and/or no stent placement following Anderson–Hynes pyeloplasty. Case reports, single‐arm studies, review articles, animal studies and experimental studies were excluded.

### Data Extraction

Data were extracted independently by one reviewer and verified by a second reviewer for accuracy and completeness. Discrepancies were resolved by consensus or consultation with a third reviewer. Extracted variables included study characteristics (first author, year of publication, country, study design, study period, and total sample size), patient demographics (age and sex), intervention details (operative technique, side of surgery, drain placement, unilateral or bilateral PUJO, operative setting) and all pre‐defined primary and secondary outcomes. Data were recorded in a standardised Excel spreadsheet (Microsoft Excel, Redmond, WA, USA).

### Risk of Bias/Quality Assessment

Risk of bias was assessed independently by two reviewers using the Cochrane Risk of Bias 2 (RoB‐2) tool for RCTs and the Risk Of Bias In Non‐randomised Studies of Interventions (ROBINS‐I) tool for non‐randomised studies of interventions [18, 19]. For ROBINS‐I, chosen pre‐specified confounding factors were age and operation technique (open, laparoscopic or robotic). Disagreements were resolved by consensus or consultation with a third reviewer.

The certainty of evidence was evaluated using the Grading of Recommendations, Assessment, Development and Evaluation (GRADE) framework [20]. The assessment considered risk of bias, inconsistency, indirectness, imprecision and potential publication bias. Based on these domains, the certainty of evidence was categorised as ‘high’, ‘moderate’, ‘low’ or ‘very low’.

### Data Synthesis

Statistical analyses were performed using R (version 4.5.1; R Foundation for Statistical Computing, Vienna, Austria). For continuous variables lacking reported means and SDs, values were estimated from medians and dispersion measures using established distribution‐based methods [21]. Composite outcomes were derived where appropriate.

To explore direct comparisons and assess heterogeneity, pairwise meta‐analyses were conducted using the ‘metafor’ package [22]. A frequentist network meta‐analysis was subsequently performed using the ‘netmeta’ package to compare all three drainage strategies simultaneously [23]. Binary outcomes were expressed as odds ratios (ORs) with 95% CIs using the Mantel–Haenszel method and continuous outcomes as mean differences (MDs) with 95% CIs. A random‐effects model was applied to account for between‐study heterogeneity. Heterogeneity was assessed using the between‐study variance (*τ* ^2^), and inconsistency was evaluated using Cochran's *Q* statistic. ‘No stent’ was defined as the reference group for all network comparisons (if available).

Network meta‐regression was performed using mean age as a covariate to evaluate its potential impact on outcomes. Analyses were stratified by study design (RCT only, non‐randomised only and combined). Results are presented as network diagrams and forest plots.

## Results

### Study Selection

The systematic literature search identified 1765 records, of which 26 studies met the pre‐defined inclusion criteria and were included in the network meta‐analysis (Table 1) [7, 8, 10, 11, 12, 13, 14, …]. These comprised five RCTs and 21 non‐randomised comparative studies. Overall, 3298 paediatric patients undergoing Anderson–Hynes pyeloplasty were included in the quantitative synthesis. The study selection process is illustrated in the PRISMA flow diagram (Fig. 1).

**Table 1:** Study characteristics.

Study	Design	*N*	Age, years, mean	JJ stent	External stent	No stent	Overall number of complications	Major complications	Minor complications	Success rate	Urinoma‐urine leakage major	Urinoma‐urine leakage minor	UTI/ pyelonephritis	Renal function	Stent‐related complication	Hospital stay (days)
Arda (2002)[24]	RCT	31	2.95		X	X	X	X	X	X		X	X		X	X
Bayne et al. (2011) [14]	Non‐RCT	367	4.68		X	X	X	X	X	X	X		X			X
Braga (2008)[25]	Non‐RCT	470	1.5	X	X		X	X	X	X		X	X		X	X
Chu (2018)[26]	Non‐RCT	61	6.9	X	X		X	X	X	X		X	X		X	X
Elmalik et al., (2008) [11]	Non‐RCT	102	4.91	X		X	X	X	X	X	X		X	X	X	X
Garg (2015)[27]	RCT	40	3.23	X	X		X	X	X	X	X	X	X		X	X
Grant (2021)[28]	Non‐RCT	310		X		X	X	X	X		X				X	
Helmy (2011)[29]	Non‐RCT	22	2	X	X		X	X	X	X					X	X
Kim et al. (2012) [10]	Non‐RCT	76	2.2	X		X	X	X	X	X			X	X		
Kocvara (2014)[30]	Non‐RCT	70	3.2	X	X	X	X	X	X	X	X		X		X	
Kong et al. (2021) [7]	Non‐RCT	650	4.45	X	X		X	X	X	X		X	X		X	X
Laddha (2019)[31]	Non‐RCT	45	3.6	X		X	X	X	X	X		X	X			X
Lee (2015)[32]	Non‐RCT	62	5.4	X	X		X	X	X	X						X
Lombardo (2021)[33]	Non‐RCT	103	6.42	X	X		X	X	X	X			X		X	X
Loulah (2018)[34]	Non‐RCT	20	6.8	X		X	X	X	X	X		X	X	X		X
Nagdeve (2018)[35]	RCT	39	4.24	X		X	X	X	X		X		X	X	X	X
Nasser et al. (2017) [8]	RCT	30	0.3		X	X	X	X	X	X	X		X	X	X	X
Noh (2016)[36]	Non‐RCT	40	6.17	X		X	X			X						X
Paraboschi (2021)[37]	Non‐RCT	53	6.88	X	X		X	X	X	X			X		X	X
Sarhan (2021)[38]	Non‐RCT	175	2.60	X	X		X	X	X	X		X	X		X	X
Siddique (2021)[39]	RCT	60	6	X		X	X	X	X	X		X				X
Smith et al. (2002) [13]	Non‐RCT	117			X	X	X	X	X	X	X	X	X		X	X
Soliman (2016)[40]	Non‐RCT	64		X		X	X	X	X	X	X		X		X	X
Son (2011)[41]	Non‐RCT	155			X	X	X	X	X	X	X	X	X			
Venturini (2019)[42]	Non‐RCT	50	8.1	X		X	X	X	X				X			X
Zoeller (2014)[43]	Non‐RCT	86	5.6	X	X		X	X	X	X	X		X		X	X

### Study Characteristics

The number of patients treated with each strategy was: JJ stent, 1630; external stent, 925; and no stent, 743. Across all patients, the pooled mean (95% CI) age was 4.42 (3.44–5.40) years. Strategy‐specific pooled mean (95% CI) ages were 5.74 (4.60–6.88) years for JJ stenting, 3.13 (2.22–4.04) years for external stenting, and 4.29 (2.67–5.92) years for no stenting. Detailed study and patient characteristics are summarised in Table 1.

### Network Geometry

For the primary outcome, overall number of complications, direct comparisons between JJ stenting, external stenting and no stenting were available, resulting in a fully connected treatment network. This network structure permitted direct and indirect comparisons between all three stent strategies for the primary outcome. For secondary outcomes, the availability of direct comparisons varied by outcome. However, all networks were sufficiently connected to allow network meta‐analysis.

### Primary Outcome: Overall Number of Complications

The overall number of complications was reported in all studies so that all 3298 patients were included in the analysis. The pooled OR (95% CI) for the overall number of complications was 0.20 (0.12–0.34), 0.14 (0.09–0.23), 0.21 (0.10–0.45) for JJ stenting, external stenting, and no stenting, respectively. In the combined network meta‐analysis, no statistically significant difference in overall complication rate was observed between external stenting and no stenting (OR 0.69, 95% CI 0.38–1.25) or between JJ stenting and no stenting (OR 0.96, 95% CI 0.55–1.68) (Fig. 2, Table 2). The difference between JJ stenting and external stenting was also not statistically significant (OR 1.40, 95% CI 0.85–2.29).

**Fig. 2:**
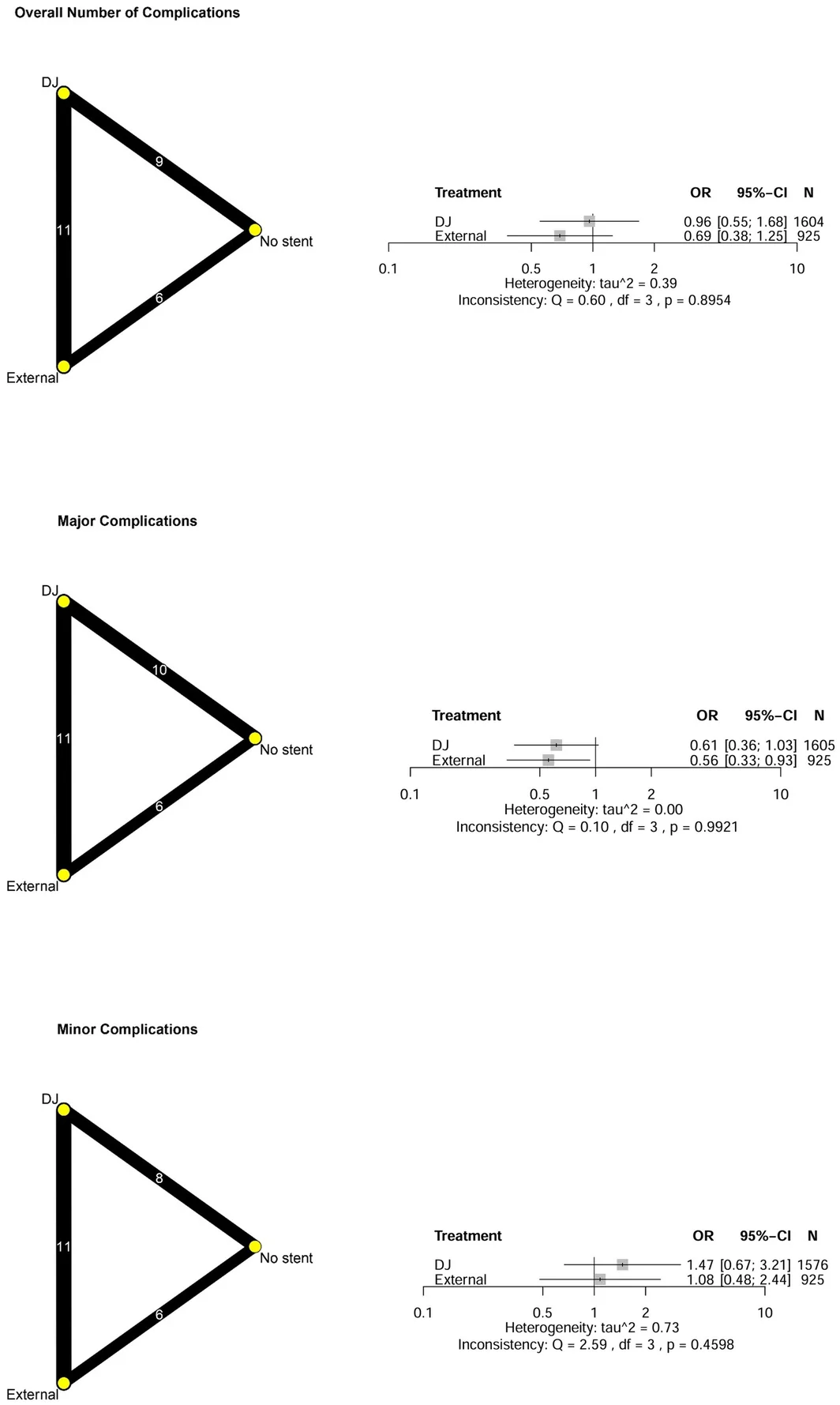
Network geometry and treatment effects for complications. Network plots show available comparisons between drainage strategies, with edge thickness reflecting the number of studies. Forest plots present network meta‐analysis results as ORs (95% CIs) using ‘no stent’ as reference for overall number of complications, number of major complications, and number of minor complications.

**Table 2:** Results of network meta‐analysis and direct comparisons of drainage strategies after paediatric pyeloplasty.

Outcome	Treatment	JJ	External	No stent
Overall number of complications, OR (95% CI)	JJ	JJ	1.51 (0.87–2.64)	0.85 (0.43–1.66)
Overall number of complications, OR (95% CI)	External	1.40 (0.85–2.29)	External	0.83 (0.38–1.81)
Overall number of complications, OR (95% CI)	No stent	0.96 (0.55–1.68)	0.69 (0.38–1.25)	No stent
Major complications, OR (95% CI)	JJ	JJ	1.12 (0.60–2.09)	0.62 (0.33–1.16)
Major complications, OR (95% CI)	External	1.10 (0.66–1.86)	External	0.56 (0.30–1.03)
Major complications, OR (95% CI)	No stent	0.61 (0.36–1.03)	0.56 (0.33–0.93)	No stent
Minor complications, OR (95% CI)	JJ	JJ	1.52 (0.75–3.06)	1.17 (0.44–3.06)
Minor complications, OR (95% CI)	External	1.35 (0.71–2.56)	External	1.49 (0.51–4.40)
Minor complications, OR (95% CI)	No stent	1.47 (0.67–3.21)	1.08 (0.48–2.44)	No stent
Operative success, OR (95% CI)	JJ	JJ	0.92 (0.51–1.65)	1.37 (0.60–3.16)
Operative success, OR (95% CI)	External	1.00 (0.59–1.69)	External	0.96 (0.40–2.29)
Operative success, OR (95% CI)	No stent	1.17 (0.61–2.25)	1.17 (0.60–2.29)	No stent
Urinoma‐urine leakage major, OR (95% CI)	JJ	JJ	0.65 (0.09–4.56)	0.23 (0.07–0.77)
Urinoma‐urine leakage major, OR (95% CI)	External	0.98 (0.25–3.79)	External	0.18 (0.05–0.60)
Urinoma‐urine leakage major, OR (95% CI)	No stent	0.20 (0.07–0.60)	0.20 (0.07–0.61)	No stent
Urinoma‐urine leakage minor, OR (95% CI)	JJ	JJ	0.40 (0.19–0.85)	0.38 (0.12–1.24)
Urinoma‐urine leakage minor, OR (95% CI)	External	0.41 (0.20–0.84)	External	0.72 (0.13–4.07)
Urinoma‐urine leakage minor, OR (95% CI)	No stent	0.35 (0.13–0.96)	0.85 (0.29–2.53)	No stent
UTI/pyelonephritis, OR (95% CI)	JJ	JJ	1.83 (0.89–3.77)	2.14 (0.89–5.15)
UTI/pyelonephritis, OR (95% CI)	External	1.64 (0.88–3.07)	External	1.74 (0.75–4.04)
UTI/pyelonephritis, OR (95% CI)	No stent	2.46 (1.23–4.93)	1.50 (0.76–2.97)	No stent
Renal function postoperative*, MD (95% CI)	JJ	JJ	.	−1.91 (−10.74 to 6.92)
Renal function postoperative*, MD (95% CI)	External	1.99 (−16.86 to 20.85)	External	−3.90 (−20.56 to 12.76)
Renal function postoperative*, MD (95% CI)	No stent	−1.91 (−10.74 to 6.92)	−3.90 (−20.56 to12.76)	No stent
Stent‐related complications, OR (95% CI)	JJ	JJ	1.46 (0.53–4.00)	1.35 (0.34–5.34)
Stent‐related complications, OR (95% CI)	External	1.41 (0.55–3.59)	External	1.11 (0.23–5.44)
Stent‐related complications, OR (95% CI)	No stent	1.41 (0.44–4.51)	1.01 (0.30–3.41)	No stent
Hospital stay, days*, MD (95% CI)	JJ	JJ	−1.46 (−2.44 to −0.47)	−0.43 (−1.54 to 0.68)
Hospital stay, days*, MD (95% CI)	External	−1.15 (−2.04 to −0.26)	External	−0.60 (−2.34 to 1.14)
Hospital stay, days*, MD (95% CI)	No stent	−0.81 (−1.78 to 0.16)	0.34 (−0.79 to 1.47)	No stent

Pairwise comparisons are presented as ORs or MDs with 95% CIs (MD marked with *). Values below the diagonal represent estimates from the network meta‐analysis, whereas values above the diagonal reflect direct pairwise comparisons. ‘No stent’ was used as the reference treatment.

When analyses were stratified by study design, estimates derived from RCTs were characterised by wide CIs and did not demonstrate statistically significant differences between strategies. Analyses restricted to non‐randomised studies yielded effect estimates in the same direction but with narrower CIs, contributing greater precision to the combined analysis. No qualitative differences in the direction of treatment effects were observed between randomised and non‐randomized evidence (Appendices S2 and S6).

### Secondary Outcomes

#### Major Complications

Major complications were reported in 25 studies comprising 3258 patients. The pooled OR (95% CI) for major complications was 0.05 (0.03–0.08), 0.06 (0.03–0.10), and 0.09 (0.05–0.16) for JJ stenting, external stenting, and no stenting, respectively. In the combined analysis, external stenting was associated with lower odds of major complications compared with no stenting (OR 0.56, 95% CI 0.33–0.93) (Fig. 2, Table 2). Differences between JJ stenting and no stenting were not statistically significant (OR 0.61, 95% CI 0.36–1.03). No statistically significant difference was observed between JJ and external stenting (OR 1.10, 95% CI 0.66–1.86). Stratified analyses by study design demonstrated consistent directions of effect (Appendix S2).

#### Minor Complications

Minor complications were reported in 25 studies comprising 3258 patients. The pooled OR (95% CI) for minor complications was 0.14 (0.07–0.25), 0.09 (0.06–0.14), and 0.08 (0.03–0.24) for JJ stenting, external stenting, and no stenting, respectively. In the combined analysis, no statistically significant differences were observed between external stenting and no stenting (OR 1.08, 95% CI 0.48–2.44) or between JJ stenting and no stenting (OR 1.47, 95% CI 0.67–3.21) (Fig. 2, Table 2). Differences between JJ and external stenting were also not statistically significant (OR 1.35, 95% CI 0.71–2.56). Results were consistent across study designs (Appendix S2).

#### Operative Success

Operative success was reported in 23 studies including 2899 patients. The pooled OR (95% CI) for operative success was 20.18 (13.52–30.13), 21.90 (15.25–31.43), and 15.17 (8.56–26.89), with a success rate of 97.1%, 97.3%, and 96.8% for JJ stenting, external stenting, and no stenting, respectively. In the combined analysis, neither external stenting (OR 1.17, 95% CI 0.60–2.29) nor JJ stenting (OR 1.17, 95% CI 0.61–2.25) were associated with higher odds of operative success compared with no stenting (Fig. 3, Table 2). No statistically significant difference was observed between JJ and external stents (OR 1.00, 95% CI 0.59–1.69). High success rates were observed across all strategies and study designs (Appendix S2).

**Fig. 3:**
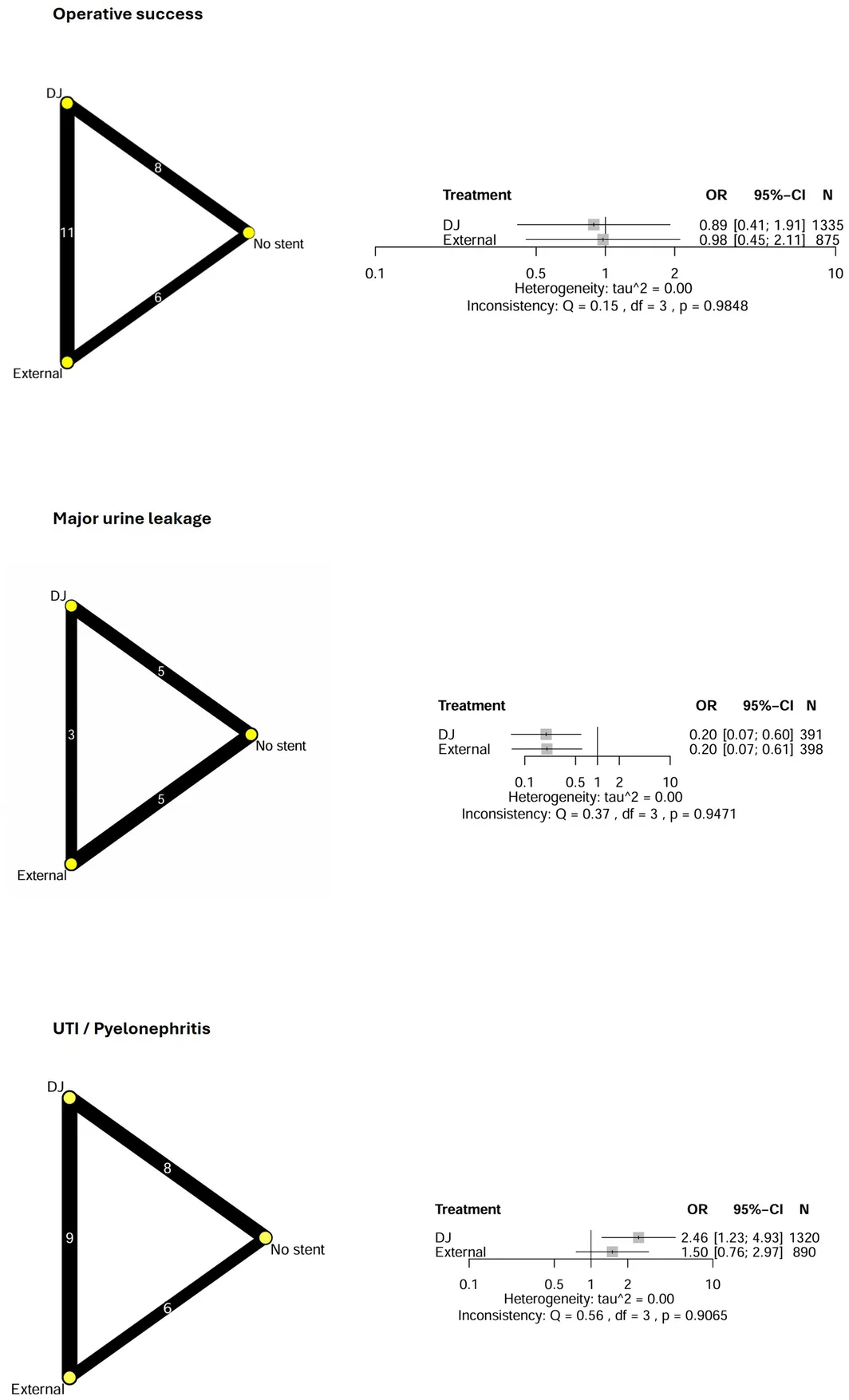
Network geometry and treatment effects for selected outcomes. Network plots show available comparisons between drainage strategies, with edge thickness reflecting the number of studies. Forest plots present network meta‐analysis results as ORs (95% CIs) using ‘no stent’ as reference for success rate, major urine leakage, and UTI/pyelonephritis.

#### Urine Leakage

Urine leakage or urinoma was pooled as one outcome termed urine leakage and stratified by severity. Major urine leakage, defined as urine leakage requiring intervention under general anaesthesia (Clavien–Dindo Grade IIIb complication), was reported in 11 studies including 1465 patients [17]. The pooled OR (95% CI) for major urine leakage was 0.02 (0.01–0.05), 0.02 (0.01–0.06), and 0.08 (0.04–0.17), with a major urine leakage rate of 0.6%, 0.6%, and 4.5% for JJ stenting, external stenting, and no stenting, respectively. For major urine leakage, external stenting was associated with lower odds compared with no stenting (OR 0.20, 95% CI 0.07–0.61) (Fig. 3, Table 2). JJ stenting similarly demonstrated a statistically significant difference compared with no stenting for major urine leakage (OR 0.20, 95% CI 0.07–0.60). JJ and external stenting did not differ significantly for major urine leakage (OR 0.98, 95% CI 0.25–3.79).

Minor urine leakage, defined as urine leakage treated conservatively, was also reported in 11 studies with 1824 patients. The pooled OR (95% CI) for minor urine leakage was 0.03 (0.01–0.07), 0.05 (0.02–0.13), and 0.09 (0.02–0.30), with a minor urine leakage rate of 1.7%, 3.0%, and 2.8% for JJ stenting, external stenting, and no stenting, respectively. For minor urine leakage our analysis showed statistically significant differences between JJ stenting and no stenting (OR 0.35, 95% CI 0.13–0.96) but not between external stenting and no stenting (OR 0.85, 95% CI 0.29–2.53). JJ stenting showed lower odds of minor urine leakage compared to external stenting (OR 0.41, 95% CI 0.20–0.84). Results were consistent across study designs (Appendix S2).

#### Urinary Tract Infection/Pyelonephritis

Postoperative UTI or pyelonephritis was reported in 22 studies including 2807 patients. The pooled OR (95% CI) for postoperative UTI or pyelonephritis was 0.07 (0.04–0.11), 0.04 (0.02–0.05), and 0.04 (0.02–0.06), with a pooled rate of 4.1%, 2.3%, and 2.6% for JJ stenting, external stenting, and no stenting, respectively. In the pooled analysis of UTI and pyelonephritis as a combined outcome, external stenting did not significantly differ from no stenting (OR 1.50, 95% CI 0.76–2.97) (Fig. 3, Table 2). JJ stenting was associated with higher odds of UTI or pyelonephritis compared with no stenting (OR 2.46, 95% CI 1.23–4.93). No statistically significant differences were observed between JJ and external stenting (OR 1.64, 95% CI 0.88–3.07). Estimates derived from RCTs were imprecise but consistent with the combined analysis (Appendix S2).

#### Renal Function

Postoperative differential renal function outcomes were reported in five studies and 266 patients. Due to heterogeneous outcome definitions and limited reporting, quantitative synthesis was limited. Available estimates are presented in Table 2, with additional analyses in the Appendices S2 and S6. The evidence network was sparse, and some direct comparisons were unavailable. Available data did not demonstrate clear differences in postoperative renal function between stent strategies (Table 2).

#### Stent‐Related Complications

Stent‐related complications were reported in 17 studies and 2426 patients. The pooled OR (95% CI) for stent‐related complications was 0.07 (0.03–0.14), 0.06 (0.03–0.13), and 0.07 (0.03–0.22), with a complication rate of 3.4%, 2.7%, and 4.7% for JJ stenting, external stenting, and no stenting, respectively. In the combined analysis, no significant differences were observed between external stenting and no stenting (OR 1.01, 95% CI 0.30–3.41), JJ stenting and no stenting (OR 1.41, 95% CI 0.44–4.51) or both stent strategies (OR 1.41, 95% CI 0.55–3.59) (Table 2).

#### Length of Hospital Stay

Length of hospital stay was reported in 22 studies including 2690 patients. The pooled mean (95% CI) for length of hospital stay was 3.73 (2.43–5.03) days, 5.13 (2.25–8.01) days, and 3.97 (2.97–4.97) days for JJ stenting, external stenting, and no stenting, respectively. In the combined analysis, no statistically significant differences were observed between external stenting and no stenting (MD 0.34, 95% CI −0.79 to 1.47 days), or between JJ stenting and no stenting (MD −0.81, 95% CI −1.78 to 0.16 days) (Table 2). Compared with external stenting, JJ stenting was associated with a statistically significantly shorter hospital stay (MD −1.15, 95% CI −2.04 to −0.26 days).

### Heterogeneity and Consistency

All analyses were conducted using random‐effects models to account for between‐study variability. Single‐arm estimates were, in some cases, highly heterogeneous (Appendix S5). Effect estimates of pairwise comparisons were also heterogeneous for some endpoints (Appendix S6). However, heterogeneity in the combined network analyses was generally low, and Cochran's *Q* statistic did not indicate relevant inconsistency within the networks for any key outcome. Moderate heterogeneity was observed for some outcomes, particularly those informed predominantly by non‐randomised studies or characterised by low event rates.

### Meta‐Regression by Age

Mean age differed substantially between studies, but also within single studies. Network meta‐regression using mean age as a covariate did not identify age as a statistically significant effect modifier for any of the key outcomes (data available upon request). Effect estimates of age on overall number of complications were 0.13 (0.17, *P* = 0.42), 0.14 (0.12, *P* = 0.25), and −0.1 (0.27, *P* = 0.7) per increase by 1 year in mean age for JJ, external, and no stenting, respectively. Inclusion of age in the models did not alter the magnitude or direction of treatment effect estimates compared with the primary analyses.

### Sensitivity Analyses

Sensitivity analyses were performed through pre‐specified stratification by study design, with network meta‐analyses conducted separately for RCTs, non‐randomised studies, and all studies combined. Across all key outcomes, the direction of effect estimates was consistent between different study designs. Estimates derived from RCTs were less precise due to smaller sample sizes and fewer events. No stratified analysis resulted in a qualitative change in conclusions compared with the combined analyses.

### Risk of Bias Assessment

Risk of bias for RCTs, assessed with the RoB‐2 tool, showed ‘some concerns’ overall in the majority of trials [18] (Fig. 4). The main limitations were related to the randomisation process and deviations from intended interventions, with a proportion of studies rated at high risk in these domains. In contrast, missing outcome data was consistently assessed as low risk across trials.

**Fig. 4:**
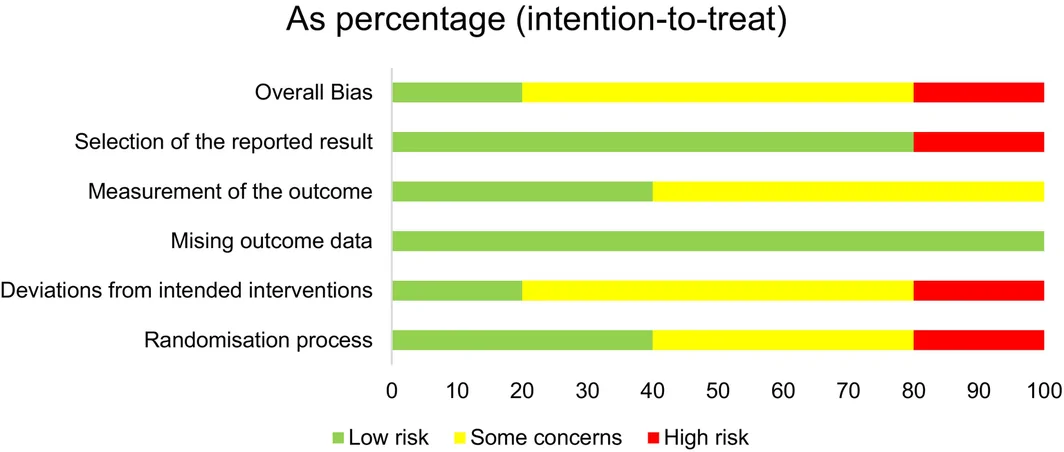
Risk of bias assessment of RCTs (RoB‐2). Proportion of studies rated as low risk (green), some concerns (yellow) and high risk (red) across RoB‐2 domains.

For non‐randomised studies, risk of bias was evaluated using the ROBINS‐I tool [19]. The majority of observational studies were judged to have serious risk of bias and four were classified as critical risk (Appendices S3 and S4). These judgements were mainly driven by concerns regarding confounding, deviations from intended interventions, measurement of outcomes, and selection of reported results. Overall, the observational evidence demonstrated substantial methodological limitations and risk of bias.

### Grade of Evidence

According to the GRADE framework, the overall certainty of evidence was judged as low for most outcomes [20]. This was primarily due to serious risk of bias in the non‐randomised studies and imprecision of effect estimates in the RCTs. For outcomes predominantly informed by observational data with serious or critical risk of bias, the certainty of evidence was considered very low.

## Discussion

This updated network meta‐analysis compared internal JJ stenting, external transanastomotic stenting, and stentless pyeloplasty in paediatric PUJO. Operative success rates were uniformly high across all drainage strategies and no clinically meaningful differences were observed. Given these consistently high success rates, operative success alone is unlikely to guide clinical decision‐making. Therefore, overall complication rate was selected as the primary outcome in this study, as differences between drainage strategies were deemed more likely to manifest in complication profiles rather than failure rates.

No statistically significant differences were detected between strategies in terms of overall complication rates. However, when complications were stratified by severity, external stenting was associated with lower odds of major complications compared with no stent placement. In contrast, minor complications did not differ significantly between strategies. Similarly, both stented approaches reduced the risk of major urine leakage or urinoma compared with stentless repair. These findings indicate that while overall complication rates appear comparable, the distribution of complication severity differs and favours a stented approach regarding clinically relevant adverse events. Differentiation between major and minor complications is critical, as major complications require invasive intervention, typically prolonging hospitalisation, whereas minor complications are frequently self‐limiting.

With respect to infectious outcomes, JJ stenting was associated with higher odds of UTI or pyelonephritis compared with no stent, whereas external stenting did not demonstrate a similar association. Although this represents a trade‐off between drainage strategies, the magnitude of this effect should be interpreted cautiously given the predominance of observational data and potential confounding. Additionally, heterogeneity in external stent management exists, as some externalised stents are clamped or tied off postoperatively and may function primarily as transanastomotic splints rather than continuous external drains, although this was inconsistently reported. Apart from infectious outcomes, differences between JJ and external stenting were not statistically significant. JJ stenting was associated with a shorter hospital stay compared with external stenting. The difference was small and the clinical relevance may vary depending on local discharge practices and perioperative protocols. It should however be acknowledged, that internal and external stents differ in procedural burden. JJ stents usually require a second operative episode with cystoscopy and general anaesthesia, whereas external stents can generally be removed in the outpatient setting. External stents require catheter care and may cause discomfort, dislodgement or leakage, while JJ placement can be technically challenging, particularly in younger children. The relative practicality of each strategy may also vary between open, laparoscopic and robotic pyeloplasty and depends on patient age, anatomy, caregiver burden, and surgeon experience.

Patient age may have influenced drainage selection. Although age was not explicitly reported as a selection criterion, patients receiving external stents were younger than those receiving JJ stents or undergoing stentless repair. Network meta‐regression using study‐level mean age did not identify age as a significant effect modifier and did not significantly alter the treatment estimates. However, age is a common clinical parameter, especially for paediatric issues and mean age differed noticeably between groups for some of the studies. Further studies should investigate this effect in more detail for a better adjustment and interpretation of the analysis results.

These findings are consistent with the prior network meta‐analysis by Liu et al. [15], which reported no differences in operative success or overall complication rates. The present study expands the evidence base through inclusion of recently published large comparative cohorts and provides a more detailed evaluation of complication severity using the Clavien–Dindo classification [17]. The separation of major and minor complications allows a more clinically meaningful interpretation and identifies differences between strategies that were not apparent when only overall complication rates were considered.

The certainty of evidence remains limited. According to the GRADE framework, overall certainty was low for most outcomes and very low for outcomes primarily informed by non‐randomised studies [20]. Although RCTs were included, they were generally small and yielded imprecise effect estimates. The findings were largely driven by observational studies, which frequently demonstrated serious or critical risk of bias, particularly due to confounding and retrospective design. Therefore, the findings should be interpreted with caution.

Several limitations should be acknowledged. Heterogeneity in outcome definitions and follow‐up duration may have influenced complication reporting. In particular, the primary outcome of ‘overall complications’ showed substantial heterogeneity, which may reflect differences in outcome definitions across studies and could challenge the assumptions underlying a random‐effects model. Unfortunately, postoperative renal function, although a key clinical outcome, was reported in only five studies, resulting in a sparse and imprecise evidence network that precluded firm conclusions regarding differences between drainage strategies.

Surgical approach (open, laparoscopic, robotic), perioperative management and antibiotic protocols were not consistently reported and may act as effect modifiers. Although the available evidence appears to relate predominantly to primary pyeloplasty, previous ipsilateral repair was incompletely reported across studies. Residual confounding cannot be excluded in non‐randomised comparisons. For example, the large difference in mean age between groups in a single study demonstrate that the choice of techniques was based on patient characteristics that could not be accounted for in this analysis. Lastly, differences in patient selection and clinical practice may limit transitivity in the network meta‐analysis.

Despite these limitations, this study represents the largest and most up‐to‐date network meta‐analysis of postoperative drainage strategies in paediatric pyeloplasty. Inclusion of recent large comparative studies substantially increases the cumulative sample size relative to previous analyses. Stratification by study design improves transparency and risk of bias and GRADE assessments enhance interpretability.

Both JJ and external stenting were associated with lower odds of major leakage‐related complications compared with stentless repair, while operative success was similar across strategies. These findings support using a stented approach. Differences between JJ and external stenting were modest and primarily related to infectious complications and procedural considerations. Given the low certainty of evidence, selection of drainage strategy should remain individualised and account for patient characteristics and institutional practice.

Future adequately powered RCTs focusing on complication outcomes, especially urine leakage, and standardised definitions are required to further determine whether one stented strategy offers a clinically relevant advantage.

## Conclusion

Operative success after paediatric pyeloplasty is high and comparable across all drainage strategies. While overall complication rates do not differ, both JJ and external stenting reduce the risk of major complications, particularly urine leakage, compared with stentless repair. Given the low certainty of evidence, drainage strategy should be individualised and further high‐quality RCTs are needed.

## Disclosure of Interests

The authors have no conflicts of interest to declare related to this work.

## Funding

Ophelia Aubert, MD, received funding from the Federal German Ministry of Research, Technology and Space, 01KG2411.

## Supporting information


**Appendix S1.** Search strategy.
**Appendix S2.** Results of network meta‐analysis and direct comparisons of drainage strategies after paediatric pyeloplasty according to study design. Pairwise comparisons are presented as ORs or MDs with 95% CIs (MDs marked with *). Values below the diagonal represent estimates from the network meta‐analysis, whereas values above the diagonal reflect direct pairwise comparisons. ‘No stent’ was used as the reference treatment.
**Appendix S3.** Risk of bias assessment summary plot of non‐randomised studies (ROBINS‐I). The figure summarises domain‐level judgements of bias. Four studies assessed as having a critical risk of bias during ROBINS‐I preliminary considerations were excluded from this figure.
**Appendix S4.** Risk of bias assessment traffic light plot of non‐randomised studies (ROBINS‐I). The figure summarises domain‐level judgements of bias. Four studies assessed as having a critical risk of bias during preliminary considerations were excluded from this figure.
**Appendix S5.** Forest plots demonstrating heterogeneity in single arms estimates. Heterogeneity shown for overall number of complications (JJ, no stent), minor complications (JJ, no stent), urine leakage minor (no stent) length of hospital stay (JJ, external, no stent).
**Appendix S6.** Network geometry and treatment effects for selected outcomes showing heterogenous effect estimates. Results shown for overall number of complications, Minor complications, renal function, and stent‐related complications.
**Appendix S7.** The PRISMA 2020 checklist.
**Appendix S8.** The PRISMA 2020 for Abstracts checklist.

## Data Availability

Detailed data will be made available by the authors upon reasonable request. Template data collection form and extracted data will be deposited on‐line in the institutional repository for Open Research Data from Heidelberg University after publication acceptance.
