# Supplementary material for: The optimal stent‐strategy in paediatric pyeloplasty: a systematic review and network meta‐analysis
Source: BJU Int. 2026 Aug 16;138(6):963–75. doi: 10.1111/bju.70423 (PMC13741106; doi:10.1111/bju.70423)
Supplement: Supplementary file 1 [file BJU-138-963-s001.docx]

**Supplementary Material**

**Appendix S1.** **Search strategy**

**PUBMED**

("child*"[Title/Abstract] OR "pediatric"[Title/Abstract] OR "paediatric"[Title/Abstract] OR "infant"[Title/Abstract] OR "neonate"[Title/Abstract])

AND ("stent*"[Title/Abstract] OR "DJ"[Title/Abstract] OR "double J"[Title/Abstract] OR "double-j"[Title/Abstract] OR "JJ"[Title/Abstract] OR "JJ-stent"[Title/Abstract])

AND ("pyeloplasty"[Title/Abstract] OR "ureteropelvic junction obstruction"[Title/Abstract] OR "UPJO"[Title/Abstract] OR "ureteral obstruction"[MeSH Terms])

12.02.2025: 417 results

**EMBASE**
('child'/exp OR 'pediatric':ti OR 'paediatric':ti OR 'infant':ti OR 'neonate':ti OR 'child':ab OR 'pediatric':ab OR 'paediatric':ab OR 'infant':ab OR 'neonate':ab)

AND ('stent'/exp OR 'DJ':ti OR 'double J':ti OR 'double-j':ti OR 'JJ':ti OR 'JJ-stent':ti OR 'stent':ab OR 'DJ':ab OR 'double J':ab OR 'double-j':ab OR 'JJ':ab OR 'JJ-stent':ab)

AND ('pyeloplasty'/exp OR 'ureteropelvic junction obstruction'/exp OR 'UPJO':ti OR 'ureteral obstruction':ti OR 'pyeloplasty':ab OR 'ureteropelvic junction obstruction':ab OR 'UPJO':ab OR 'ureteral obstruction':ab)

12.02.2025: 925 results

**COCHRANE**

"child" OR "pediatric" OR "paediatric" OR "infant" OR "neonate"

AND "stent" OR "DJ" OR "double J" OR "double-j" OR "JJ" OR "JJ-stent"

AND "pyeloplasty" OR "ureteropelvic junction obstruction" OR "UPJO" OR "ureteral obstruction"

12.02.2025: 36 results

**WEB OF SCIENCE**

TS=("child*" OR "pediatric" OR "paediatric" OR "infant" OR "neonate")

AND TS=("stent*" OR "DJ" OR "double J" OR "double-j" OR "JJ" OR "JJ-stent")

AND TS=("pyeloplasty" OR "ureteropelvic junction obstruction" OR "UPJO" OR "ureteral obstruction")

12.02.2025: 387 results

**Appendix S2. Results of network meta-analysis and direct comparisons of drainage strategies after pediatric pyeloplasty according to study design.** Pairwise comparisons are presented as odds ratios or mean differences with 95% confidence intervals (Mean differences marked with *). Values below the diagonal represent estimates from the network meta-analysis, whereas values above the diagonal reflect direct pairwise comparisons. “No stent” was used as the reference treatment.

| **Outcome** | **Subset** | **Treatment** | **DJ** | **External** | **No stent** |
| --- | --- | --- | --- | --- | --- |
| Overall number of complications | RCT | DJ | DJ | 23.22 [0.32, 1699.39] | 0.13 [0.00, 6.67] |
| Overall number of complications | RCT | External | 3.22 [0.13, 80.68] | External | 0.54 [0.03, 9.96] |
| Overall number of complications | RCT | No stent | 0.69 [0.03, 15.62] | 0.21 [0.02, 2.92] | No stent |
| Overall number of complications | Non-RCT | DJ | DJ | 1.33 [0.84, 2.09] | 1.16 [0.64, 2.11] |
| Overall number of complications | Non-RCT | External | 1.31 [0.87, 1.98] | External | 0.94 [0.48, 1.88] |
| Overall number of complications | Non-RCT | No stent | 1.20 [0.74, 1.96] | 0.91 [0.55, 1.53] | No stent |
| Overall number of complications | All | DJ | DJ | 1.51 [0.87, 2.64] | 0.85 [0.43, 1.66] |
| Overall number of complications | All | External | 1.40 [0.85, 2.29] | External | 0.83 [0.38, 1.81] |
| Overall number of complications | All | No stent | 0.96 [0.55, 1.68] | 0.69 [0.38, 1.25] | No stent |
| Major complications | RCT | DJ | DJ | 0.63 [0.09, 4.24] | 0.82 [0.08, 8.28] |
| Major complications | RCT | External | 1.07 [0.22, 5.21] | External | 0.24 [0.05, 1.23] |
| Major complications | RCT | No stent | 0.38 [0.07, 2.07] | 0.35 [0.08, 1.49] | No stent |
| Major complications | Non-RCT | DJ | DJ | 1.20 [0.62, 2.33] | 0.60 [0.31, 1.17] |
| Major complications | Non-RCT | External | 1.09 [0.63, 1.90] | External | 0.64 [0.33, 1.25] |
| Major complications | Non-RCT | No stent | 0.65 [0.37, 1.12] | 0.59 [0.34, 1.03] | No stent |
| Major complications | All | DJ | DJ | 1.12 [0.60, 2.09] | 0.62 [0.33, 1.16] |
| Major complications | All | External | 1.10 [0.66, 1.86] | External | 0.56 [0.30, 1.03] |
| Major complications | All | No stent | 0.61 [0.36, 1.03] | 0.56 [0.33, 0.93] | No stent |
| Minor complications | RCT | DJ | DJ | 13.22 [0.08, 2150.41] | 0.13 [0.00, 20.23] |
| Minor complications | RCT | External | 1.77 [0.03, 94.40] | External | 1.76 [0.03, 88.76] |
| Minor complications | RCT | No stent | 0.94 [0.02, 49.18] | 0.53 [0.02, 16.60] | No stent |
| Minor complications | Non-RCT | DJ | DJ | 1.22 [0.71, 2.08] | 2.03 [0.83, 4.96] |
| Minor complications | Non-RCT | External | 1.25 [0.76, 2.06] | External | 1.44 [0.58, 3.61] |
| Minor complications | Non-RCT | No stent | 1.91 [0.95, 3.83] | 1.52 [0.76, 3.06] | No stent |
| Minor complications | All | DJ | DJ | 1.52 [0.75, 3.06] | 1.17 [0.44, 3.06] |
| Minor complications | All | External | 1.35 [0.71, 2.56] | External | 1.49 [0.51, 4.40] |
| Minor complications | All | No stent | 1.47 [0.67, 3.21] | 1.08 [0.48, 2.44] | No stent |
| Operative success | RCT | DJ | DJ | 1.00 [0.02, 52.85] | 4.11 [0.87, 19.41] |
| Operative success | RCT | External | 2.99 [0.41, 21.83] | External | 0.95 [0.17, 5.19] |
| Operative success | RCT | No stent | 3.48 [0.81, 14.98] | 1.16 [0.24, 5.62] | No stent |
| Operative success | Non-RCT | DJ | DJ | 0.91 [0.50, 1.66] | 0.88 [0.33, 2.37] |
| Operative success | Non-RCT | External | 0.91 [0.52, 1.58] | External | 0.96 [0.35, 2.65] |
| Operative success | Non-RCT | No stent | 0.89 [0.41, 1.91] | 0.98 [0.45, 2.11] | No stent |
| Operative success | All | DJ | DJ | 0.92 [0.51, 1.65] | 1.37 [0.60, 3.16] |
| Operative success | All | External | 1.00 [0.59, 1.69] | External | 0.96 [0.40, 2.29] |
| Operative success | All | No stent | 1.17 [0.61, 2.25] | 1.17 [0.60, 2.29] | No stent |
| Urine leakage major | RCT | DJ | DJ | 0.18 [0.01, 4.01] | . |
| Urine leakage major | RCT | External | 0.18 [0.01, 4.01] | External | 0.05 [0.00, 0.94] |
| Urine leakage major | RCT | No stent | 0.01 [0.00, 0.63] | 0.05 [0.00, 0.94] | No stent |
| Urine leakage major | Non-RCT | DJ | DJ | 1.50 [0.12, 18.38] | 0.16 [0.04, 0.62] |
| Urine leakage major | Non-RCT | External | 0.81 [0.16, 4.15] | External | 0.29 [0.07, 1.14] |
| Urine leakage major | Non-RCT | No stent | 0.19 [0.05, 0.69] | 0.24 [0.07, 0.85] | No stent |
| Urine leakage major | All | DJ | DJ | 0.65 [0.09, 4.56] | 0.16 [0.04, 0.62] |
| Urine leakage major | All | External | 0.70 [0.17, 2.86] | External | 0.21 [0.06, 0.73] |
| Urine leakage major | All | No stent | 0.15 [0.05, 0.51] | 0.22 [0.07, 0.67] | No stent |
| Urine leakage minor | RCT | DJ | DJ | 0.09 [0.00, 1.78] | . |
| Urine leakage minor | RCT | External | 0.09 [0.00, 1.78] | External | 1.07 [0.06, 18.82] |
| Urine leakage minor | RCT | No stent | 0.10 [0.00, 6.04] | 1.07 [0.06, 18.82] | No stent |
| Urine leakage minor | Non-RCT | DJ | DJ | 0.16 [0.02, 1.07] | 0.00 [0.00, 0.24] |
| Urine leakage minor | Non-RCT | External | 0.11 [0.02, 0.64] | External | 0.58 [0.04, 8.96] |
| Urine leakage minor | Non-RCT | No stent | 0.03 [0.00, 0.40] | 0.25 [0.02, 2.74] | No stent |
| Urine leakage minor | All | DJ | DJ | 0.17 [0.04, 0.69] | 0.00 [0.00, 0.17] |
| Urine leakage minor | All | External | 0.12 [0.03, 0.47] | External | 0.72 [0.10, 5.21] |
| Urine leakage minor | All | No stent | 0.05 [0.01, 0.39] | 0.39 [0.06, 2.36] | No stent |
| Urinoma major | RCT | DJ | DJ | . | 1.06 [0.06, 18.17] |
| Urinoma major | RCT | No stent | 1.06 [0.06, 18.17] | . | No stent |
| Urinoma major | Non-RCT | External | . | External | 0.17 [0.01, 3.37] |
| Urinoma major | Non-RCT | No stent | . | 0.17 [0.01, 3.37] | No stent |
| Urinoma major | All | DJ | DJ | . | 1.06 [0.06, 18.17] |
| Urinoma major | All | External | 6.21 [0.10, 383.90] | External | 0.17 [0.01, 3.37] |
| Urinoma major | All | No stent | 1.06 [0.06, 18.17] | 0.17 [0.01, 3.37] | No stent |
| Urinoma minor | RCT | DJ | DJ | . | 0.41 [0.11, 1.50] |
| Urinoma minor | RCT | No stent | 0.41 [0.11, 1.50] | . | No stent |
| Urinoma minor | Non-RCT | DJ | DJ | 1.39 [0.34, 5.76] | 0.28 [0.03, 2.45] |
| Urinoma minor | Non-RCT | External | 1.39 [0.34, 5.76] | External | . |
| Urinoma minor | Non-RCT | No stent | 0.28 [0.03, 2.45] | 0.20 [0.02, 2.68] | No stent |
| Urinoma minor | All | DJ | DJ | 1.39 [0.34, 5.76] | 0.37 [0.12, 1.13] |
| Urinoma minor | All | External | 1.39 [0.34, 5.76] | External | . |
| Urinoma-Urine leakage major | RCT | DJ | DJ | 0.18 [0.00, 45.14] | 1.06 [0.00, 229.75] |
| Urinoma-Urine leakage major | RCT | External | 0.94 [0.01, 82.70] | External | 0.05 [0.00, 11.08] |
| Urinoma-Urine leakage major | RCT | No stent | 0.22 [0.00, 18.42] | 0.24 [0.00, 20.30] | No stent |
| Urinoma-Urine leakage major | Non-RCT | DJ | DJ | 1.50 [0.12, 18.38] | 0.16 [0.04, 0.62] |
| Urinoma-Urine leakage major | Non-RCT | External | 0.95 [0.19, 4.82] | External | 0.23 [0.06, 0.89] |
| Urinoma-Urine leakage major | Non-RCT | No stent | 0.19 [0.05, 0.66] | 0.20 [0.06, 0.69] | No stent |
| Urinoma-Urine leakage major | All | DJ | DJ | 0.65 [0.09, 4.56] | 0.23 [0.07, 0.77] |
| Urinoma-Urine leakage major | All | External | 0.98 [0.25, 3.79] | External | 0.18 [0.05, 0.60] |
| Urinoma-Urine leakage major | All | No stent | 0.20 [0.07, 0.60] | 0.20 [0.07, 0.61] | No stent |
| Urinoma-Urine leakage minor | RCT | DJ | DJ | 0.09 [0.00, 1.78] | 0.41 [0.11, 1.50] |
| Urinoma-Urine leakage minor | RCT | External | 0.18 [0.02, 1.56] | External | 1.07 [0.06, 18.82] |
| Urinoma-Urine leakage minor | RCT | No stent | 0.36 [0.11, 1.24] | 2.02 [0.24, 17.41] | No stent |
| Urinoma-Urine leakage minor | Non-RCT | DJ | DJ | 0.44 [0.20, 0.97] | 0.26 [0.02, 4.66] |
| Urinoma-Urine leakage minor | Non-RCT | External | 0.45 [0.21, 0.95] | External | 0.58 [0.07, 5.04] |
| Urinoma-Urine leakage minor | Non-RCT | No stent | 0.26 [0.04, 1.57] | 0.58 [0.10, 3.36] | No stent |
| Urinoma-Urine leakage minor | All | DJ | DJ | 0.40 [0.19, 0.85] | 0.38 [0.12, 1.24] |
| Urinoma-Urine leakage minor | All | External | 0.41 [0.20, 0.84] | External | 0.72 [0.13, 4.07] |
| Urinoma-Urine leakage minor | All | No stent | 0.35 [0.13, 0.96] | 0.85 [0.29, 2.53] | No stent |
| UTI / Pyelonephritis | RCT | DJ | DJ | 3.15 [0.12, 82.16] | 6.79 [0.71, 64.72] |
| UTI / Pyelonephritis | RCT | External | 4.57 [0.46, 45.35] | External | 1.03 [0.10, 10.49] |
| UTI / Pyelonephritis | RCT | No stent | 5.69 [0.80, 40.55] | 1.25 [0.17, 9.21] | No stent |
| UTI / Pyelonephritis | Non-RCT | DJ | DJ | 1.77 [0.84, 3.73] | 1.74 [0.67, 4.51] |
| UTI / Pyelonephritis | Non-RCT | External | 1.51 [0.79, 2.89] | External | 1.88 [0.76, 4.65] |
| UTI / Pyelonephritis | Non-RCT | No stent | 2.22 [1.05, 4.69] | 1.47 [0.71, 3.06] | No stent |
| UTI / Pyelonephritis | All | DJ | DJ | 1.83 [0.89, 3.77] | 2.14 [0.89, 5.15] |
| UTI / Pyelonephritis | All | External | 1.64 [0.88, 3.07] | External | 1.74 [0.75, 4.04] |
| UTI / Pyelonephritis | All | No stent | 2.46 [1.23, 4.93] | 1.50 [0.76, 2.97] | No stent |
| UTI | RCT | DJ | DJ | 3.15 [0.12, 82.16] | 6.79 [0.71, 64.72] |
| UTI | RCT | External | 4.57 [0.46, 45.35] | External | 1.03 [0.10, 10.49] |
| UTI | RCT | No stent | 5.69 [0.80, 40.55] | 1.25 [0.17, 9.21] | No stent |
| UTI | Non-RCT | DJ | DJ | 1.84 [0.75, 4.50] | 1.57 [0.58, 4.27] |
| UTI | Non-RCT | External | 1.46 [0.69, 3.07] | External | 1.87 [0.74, 4.72] |
| UTI | Non-RCT | No stent | 2.11 [0.96, 4.64] | 1.45 [0.68, 3.11] | No stent |
| UTI | All | DJ | DJ | 1.91 [0.81, 4.53] | 2.00 [0.80, 4.97] |
| UTI | All | External | 1.63 [0.80, 3.32] | External | 1.72 [0.73, 4.07] |
| UTI | All | No stent | 2.39 [1.15, 4.96] | 1.47 [0.72, 2.98] | No stent |
| Pyelonephritis | Non-RCT | DJ | DJ | 1.64 [0.43, 6.29] | 5.05 [0.19, 131.55] |
| Pyelonephritis | Non-RCT | External | 1.64 [0.43, 6.29] | External | 2.23 [0.04, 118.69] |
| Pyelonephritis | Non-RCT | No stent | 4.64 [0.20, 110.11] | 2.83 [0.10, 76.84] | No stent |
| Pyelonephritis | All | DJ | DJ | 1.64 [0.43, 6.29] | 5.05 [0.19, 131.55] |
| Pyelonephritis | All | External | 1.64 [0.43, 6.29] | External | 2.23 [0.04, 118.69] |
| Pyelonephritis | All | No stent | 4.64 [0.20, 110.11] | 2.83 [0.10, 76.84] | No stent |
| Renal function postoperative * | RCT | DJ | DJ | . | 1.98 [ -0.92, 4.88] |
| Renal function postoperative * | RCT | External | 5.88 [ -1.75, 13.51] | External | -3.90 [-10.96, 3.16] |
| Renal function postoperative * | RCT | No stent | 1.98 [ -0.92, 4.88] | -3.90 [-10.96, 3.16] | No stent |
| Renal function postoperative * | Non-RCT | DJ | DJ | . | -3.82 [-15.48, 7.85] |
| Renal function postoperative * | Non-RCT | No stent | -3.82 [-15.48, 7.85] | . | No stent |
| Renal function postoperative * | All | DJ | DJ | . | -1.91 [-10.74, 6.92] |
| Renal function postoperative * | All | External | 1.99 [-16.86, 20.85] | External | -3.90 [-20.56, 12.76] |
| Renal function postoperative * | All | No stent | -1.91 [-10.74, 6.92] | -3.90 [-20.56, 12.76] | No stent |
| Stent-related complications | RCT | DJ | DJ | 1.00 [0.06, 16.13] | 3.32 [0.06, 180.84] |
| Stent-related complications | RCT | External | 1.65 [0.15, 17.98] | External | 0.49 [0.05, 5.25] |
| Stent-related complications | RCT | No stent | 1.18 [0.08, 17.40] | 0.71 [0.08, 5.96] | No stent |
| Stent-related complications | Non-RCT | DJ | DJ | 1.59 [0.50, 5.06] | 1.17 [0.24, 5.65] |
| Stent-related complications | Non-RCT | External | 1.32 [0.43, 4.01] | External | 2.56 [0.24, 27.26] |
| Stent-related complications | Non-RCT | No stent | 1.62 [0.38, 6.81] | 1.22 [0.24, 6.19] | No stent |
| Stent-related complications | All | DJ | DJ | 1.46 [0.53, 4.00] | 1.35 [0.34, 5.34] |
| Stent-related complications | All | External | 1.41 [0.55, 3.59] | External | 1.11 [0.23, 5.44] |
| Stent-related complications | All | No stent | 1.41 [0.44, 4.51] | 1.01 [0.30, 3.41] | No stent |
| Hospital stay (days)* | RCT | DJ | DJ | -6.80 [-12.41, -1.19] | -0.39 [ -4.12, 3.33] |
| Hospital stay (days)* | RCT | External | -3.11 [-6.96, 0.74] | External | -0.59 [ -4.36, 3.19] |
| Hospital stay (days)* | RCT | No stent | -2.03 [-5.29, 1.24] | 1.08 [-2.21, 4.38] | No stent |
| Hospital stay (days)* | Non-RCT | DJ | DJ | -1.01 [-2.03, 0.01] | -0.44 [-1.74, 0.86] |
| Hospital stay (days)* | Non-RCT | External | -0.91 [-1.88, 0.06] | External | -0.50 [-3.33, 2.33] |
| Hospital stay (days)* | Non-RCT | No stent | -0.61 [-1.80, 0.58] | 0.30 [-1.13, 1.72] | No stent |
| Hospital stay (days)* | All | DJ | DJ | -1.46 [-2.44, -0.47] | -0.43 [-1.54, 0.68] |
| Hospital stay (days)* | All | External | -1.15 [-2.04, -0.26] | External | -0.60 [-2.34, 1.14] |
| Hospital stay (days)* | All | No stent | -0.81 [-1.78, 0.16] | 0.34 [-0.79, 1.47] | No stent |

**Appendix S3. Risk of bias assessment summary plot of non-randomized studies (ROBINS-I).** The figure summarizes domain-level judgments of bias. Four studies assessed as having a critical risk of bias during ROBINS-I preliminary considerations were excluded from this figure.

**Appendix S4. Risk of bias assessment traffic light plot of non-randomized studies (ROBINS-I).** The figure summarizes domain-level judgments of bias. Four studies assessed as having a critical risk of bias during preliminary considerations were excluded from this figure.

**Appendix S5. Forest plots demonstrating heterogeneity in single arms estimates.** Heterogeneity shown for overall number of complications (DJ, no stent), minor complications (DJ, no stent), urine leakage minor (no stent) length of hospital stay (DJ, external, no stent).

**Appendix S6.** **Network geometry and treatment effects for selected outcomes showing heterogenous effect estimates**. Results shown for overall number of complications, Minor complications, Renal function, Stent-related complications).

**Overall number of complications**

**Minor complications**

**Renal function**

**Stent-related complications**

**Appendix S7. PRISMA 2020 Checklist**

| **Section and Topic** | **Item #** | **Checklist item** | **Location where item is reported** |
| --- | --- | --- | --- |
| **TITLE** | | |  |
| Title | 1 | Identify the report as a systematic review. | Title page, p. 1 |
| **ABSTRACT** | | |  |
| Abstract | 2 | See the PRISMA 2020 for Abstracts checklist. | Abstract, p. 2, separate Checklist filled out |
| **INTRODUCTION** | | |  |
| Rationale | 3 | Describe the rationale for the review in the context of existing knowledge. | Introduction, pp. 3-4 |
| Objectives | 4 | Provide an explicit statement of the objective(s) or question(s) the review addresses. | Introduction, p. 4 |
| **METHODS** | | |  |
| Eligibility criteria | 5 | Specify the inclusion and exclusion criteria for the review and how studies were grouped for the syntheses. | Selection criteria, pp. 5-6; Table 1, pp. 24-25 |
| Information sources | 6 | Specify all databases, registers, websites, organisations, reference lists and other sources searched or consulted to identify studies. Specify the date when each source was last searched or consulted. | Search Strategy, p. 5; Appendix S1 |
| Search strategy | 7 | Present the full search strategies for all databases, registers and websites, including any filters and limits used. | Search Strategy, p. 5; Appendix S1 |
| Selection process | 8 | Specify the methods used to decide whether a study met the inclusion criteria of the review, including how many reviewers screened each record and each report retrieved, whether they worked independently, and if applicable, details of automation tools used in the process. | Search Strategy, p. 5; Figure 1, p. 20 |
| Data collection process | 9 | Specify the methods used to collect data from reports, including how many reviewers collected data from each report, whether they worked independently, any processes for obtaining or confirming data from study investigators, and if applicable, details of automation tools used in the process. | Data extraction, p. 6 |
| Data items | 10a | List and define all outcomes for which data were sought. Specify whether all results that were compatible with each outcome domain in each study were sought (e.g. for all measures, time points, analyses), and if not, the methods used to decide which results to collect. | Selection criteria, pp. 5-6 |
|  | 10b | List and define all other variables for which data were sought (e.g. participant and intervention characteristics, funding sources). Describe any assumptions made about any missing or unclear information. | Data extraction, p. 6; Study Characteristics, p. 8; Table 1, pp. 24-25 |
| Study risk of bias assessment | 11 | Specify the methods used to assess risk of bias in the included studies, including details of the tool(s) used, how many reviewers assessed each study and whether they worked independently, and if applicable, details of automation tools used in the process. | Risk of Bias / Quality Assessment, pp. 6-7 |
| Effect measures | 12 | Specify for each outcome the effect measure(s) (e.g. risk ratio, mean difference) used in the synthesis or presentation of results. | Data synthesis, p. 7 |
| Synthesis methods | 13a | Describe the processes used to decide which studies were eligible for each synthesis (e.g. tabulating the study intervention characteristics and comparing against the planned groups for each synthesis (item #5)). | Selection criteria, pp. 5-6; Data synthesis, p. 7; Table 1, pp. 24-25 |
|  | 13b | Describe any methods required to prepare the data for presentation or synthesis, such as handling of missing summary statistics, or data conversions. | Data synthesis, p. 7 |
|  | 13c | Describe any methods used to tabulate or visually display results of individual studies and syntheses. | Data synthesis, p. 7; Figures 1-4, pp. 20-23; Tables 1-2, pp. 24-26 |
|  | 13d | Describe any methods used to synthesize results and provide a rationale for the choice(s). If meta-analysis was performed, describe the model(s), method(s) to identify the presence and extent of statistical heterogeneity, and software package(s) used. | Data synthesis, p. 7 |
|  | 13e | Describe any methods used to explore possible causes of heterogeneity among study results (e.g. subgroup analysis, meta-regression). | Data synthesis, p. 7; Heterogeneity and Consistency / Meta-regression by Age, pp. 12-13 |
|  | 13f | Describe any sensitivity analyses conducted to assess robustness of the synthesized results. | Data synthesis, p. 7; Sensitivity Analyses, p. 13; Appendix S2 |
| Reporting bias assessment | 14 | Describe any methods used to assess risk of bias due to missing results in a synthesis (arising from reporting biases). | Risk of bias / Quality assessment p 6-7 |
| Certainty assessment | 15 | Describe any methods used to assess certainty (or confidence) in the body of evidence for an outcome. | Risk of Bias / Quality Assessment, pp. 6-7 |
| **RESULTS** | | |  |
| Study selection | 16a | Describe the results of the search and selection process, from the number of records identified in the search to the number of studies included in the review, ideally using a flow diagram. | Study Selection, p. 8; Figure 1, p. 20 |
|  | 16b | Cite studies that might appear to meet the inclusion criteria, but which were excluded, and explain why they were excluded. | Figure 1, p. 20 (exclusion categories reported; individual excluded studies not cited) |
| Study characteristics | 17 | Cite each included study and present its characteristics. | Study Characteristics, p. 8; Table 1, pp. 24-25; included studies in References, pp. 18-19 |
| Risk of bias in studies | 18 | Present assessments of risk of bias for each included study. | Risk of Bias Assessment, p. 13; Figure 4, p. 23; Appendix S3-4 |
| Results of individual studies | 19 | For all outcomes, present, for each study: (a) summary statistics for each group (where appropriate) and (b) an effect estimate and its precision (e.g. confidence/credible interval), ideally using structured tables or plots. | Figures 2-3, pp. 21-22; Table 2, p. 26; Appendix S2, 5&6 |
| Results of syntheses | 20a | For each synthesis, briefly summarise the characteristics and risk of bias among contributing studies. | Study Selection / Study Characteristics, p. 8; Risk of Bias Assessment, p. 13 |
|  | 20b | Present results of all statistical syntheses conducted. If meta-analysis was done, present for each the summary estimate and its precision (e.g. confidence/credible interval) and measures of statistical heterogeneity. If comparing groups, describe the direction of the effect. | Results, pp. 8-12; Table 2, p. 26; Figures 2-3, pp. 21-22, Appendix S2 and 5-6, pp. 29-39 |
|  | 20c | Present results of all investigations of possible causes of heterogeneity among study results. | Heterogeneity and Consistency / Meta-regression by Age, pp. 12-13; Appendix S5-6 |
|  | 20d | Present results of all sensitivity analyses conducted to assess the robustness of the synthesized results. | Sensitivity Analyses, p. 13; Appendix S2 |
| Reporting biases | 21 | Present assessments of risk of bias due to missing results (arising from reporting biases) for each synthesis assessed. | Figure 4, p.23, Appendix S3&4 |
| Certainty of evidence | 22 | Present assessments of certainty (or confidence) in the body of evidence for each outcome assessed. | Grade of Evidence, p. 13 |
| **DISCUSSION** | | |  |
| Discussion | 23a | Provide a general interpretation of the results in the context of other evidence. | Discussion, pp. 14-15 |
|  | 23b | Discuss any limitations of the evidence included in the review. | Discussion, pp. 15-16 |
|  | 23c | Discuss any limitations of the review processes used. | Discussion, p. 16 |
|  | 23d | Discuss implications of the results for practice, policy, and future research. | Discussion, pp. 16-17; Conclusion, p. 17 |
| **OTHER INFORMATION** | | |  |
| Registration and protocol | 24a | Provide registration information for the review, including register name and registration number, or state that the review was not registered. | Methods, p. 5 |
|  | 24b | Indicate where the review protocol can be accessed, or state that a protocol was not prepared. | Protocol currently under review |
|  | 24c | Describe and explain any amendments to information provided at registration or in the protocol. | NA |
| Support | 25 | Describe sources of financial or non-financial support for the review, and the role of the funders or sponsors in the review. | Funding, p. 17 |
| Competing interests | 26 | Declare any competing interests of review authors. | Conflict of interest, p. 17 |
| Availability of data, code and other materials | 27 | Report which of the following are publicly available and where they can be found: template data collection forms; data extracted from included studies; data used for all analyses; analytic code; any other materials used in the review. | Data Sharing and Data Accessibility, p. 17 |

*From:* Page MJ, McKenzie JE, Bossuyt PM, Boutron I, Hoffmann TC, Mulrow CD, et al. The PRISMA 2020 statement: an updated guideline for reporting systematic reviews. BMJ 2021;372:n71. doi: 10.1136/bmj.n71. This work is licensed under CC BY 4.0. To view a copy of this license, visit <https://creativecommons.org/licenses/by/4.0/>

**Appendix S8. PRISMA 2020 for Abstracts Checklist**

| **Section and Topic** | **Item #** | **Checklist item** | **Reported (Yes/No)** |
| --- | --- | --- | --- |
| **TITLE** | | |  |
| Title | 1 | Identify the report as a systematic review. | Yes |
| **BACKGROUND** | | |  |
| Objectives | 2 | Provide an explicit statement of the main objective(s) or question(s) the review addresses. | Yes |
| **METHODS** | | |  |
| Eligibility criteria | 3 | Specify the inclusion and exclusion criteria for the review. | Yes |
| Information sources | 4 | Specify the information sources (e.g. databases, registers) used to identify studies and the date when each was last searched. | Yes |
| Risk of bias | 5 | Specify the methods used to assess risk of bias in the included studies. | Yes |
| Synthesis of results | 6 | Specify the methods used to present and synthesise results. | Yes |
| **RESULTS** | | |  |
| Included studies | 7 | Give the total number of included studies and participants and summarise relevant characteristics of studies. | Yes |
| Synthesis of results | 8 | Present results for main outcomes, preferably indicating the number of included studies and participants for each. If meta-analysis was done, report the summary estimate and confidence/credible interval. If comparing groups, indicate the direction of the effect (i.e. which group is favoured). | Yes |
| **DISCUSSION** | | |  |
| Limitations of evidence | 9 | Provide a brief summary of the limitations of the evidence included in the review (e.g. study risk of bias, inconsistency and imprecision). | Yes |
| Interpretation | 10 | Provide a general interpretation of the results and important implications. | Yes |
| **OTHER** | | |  |
| Funding | 11 | Specify the primary source of funding for the review. | No |
| Registration | 12 | Provide the register name and registration number. | No |

*From:* Page MJ, McKenzie JE, Bossuyt PM, Boutron I, Hoffmann TC, Mulrow CD, et al. The PRISMA 2020 statement: an updated guideline for reporting systematic reviews. BMJ 2021;372:n71. doi: 10.1136/bmj.n71. This work is licensed under CC BY 4.0. To view a copy of this license, visit <https://creativecommons.org/licenses/by/4.0/>
